# Addressing Risks of Violence against Healthcare Staff in Emergency Departments: The Effects of Job Satisfaction and Attachment Style

**DOI:** 10.1155/2019/5430870

**Published:** 2019-05-28

**Authors:** Sabrina Berlanda, Monica Pedrazza, Marta Fraizzoli, Federica de Cordova

**Affiliations:** Department of Human Sciences, University of Verona, via San Francesco 22, 37129 Verona, Italy

## Abstract

Violence in the workplace is one of the most serious issues affecting the healthcare sector. The incidence of violent behaviour towards healthcare workers is increasing worldwide. It is difficult to assess the extent of the problem, however, as violent incidents are underreported. In fact, many doctors and nurses see violence—perpetrated primarily by patients and visitors (friends and relatives of patients)—as a part of their job. Several studies indicate that violent behaviour against healthcare workers has serious consequences for the professionals involved, as well as for the wider healthcare system. The purpose of this study was to ascertain the prevalence of patient and visitor violence in a number of emergency departments in northeastern Italy and to explore the relationship between violence and certain psychosocial factors (adult attachment style, age, and job satisfaction). Data were collected using an online questionnaire. Our results demonstrate that patient and visitor violence in emergency departments is a serious risk for nurses and doctors and that it is affected by several factors relating to both patient pathologies and the way the workplace and work patterns are organised. Previous studies indicate that the most common form of violence experienced in these contexts is emotional violence and that nurses are more likely than doctors to suffer emotional and physical violence. Based on multiple regression analysis of the data, it appears that greater age and higher scores in secure attachment are associated with reduced experience of emotional violence from patients and visitors. Furthermore, our results show that the relationship between secure attachment and the amount of patient-and-visitor-perpetrated emotional violence experienced is mediated by levels of job satisfaction. We also discuss the potential implications of these results in terms of using staff training to prevent and manage patient and visitor violence and improve the safety of healthcare professionals.

## 1. Introduction

Recent years have seen an increase in the number of violent acts recorded around the world [1]. While representations of violence in leisure and entertainment settings, on the roads, in public spaces, and within the family are relatively common, the presence of violence in healthcare environments is an unfamiliar concept to many [2]. Unfortunately, data from the European Agency for Safety and Health at Work indicate that healthcare is actually the sector that experiences the highest rates of workplace violence [3–5]. Workplace violence can mean a single event or a number of small, recurrent incidents that, accumulatively, have the potential to cause serious harm to the worker [6]. For healthcare workers, violence represents a complex and dangerous occupational hazard [7]. The primary purpose of any healthcare system is the promotion of health while ensuring equal opportunities [8]. Patients need a calm, stable environment in which they are supported and feel safe; a healthcare setting cannot be a “battlefield” [2]. Nonetheless, the incidence of violence in the healthcare system is on the rise [9] and, while this phenomenon has been explored in particular in relation to the nursing profession [10], there is a lack of research about violence affecting doctors [11]. Doctors play a key role in healthcare systems, so it is important to study the prevalence and nature of their exposure to workplace violence [12].

The terms “violence” and “aggression” are not easily distinguished from one another. For this reason, in our study, they are treated as synonymous [13]. Furthermore, many different instruments have been developed for assessing workplace violence [14], so it can be difficult to compare different studies [15]. Another problem is the lack of uniform definitions of workplace violence in the literature [14, 16]. World Health Organization (WHO) defines violence as the intentional use of threatened or actual force against a person or a group which may cause physical or psychological trauma [17]. The European Commission defines workplace violence as “all situations when a worker is offended, threatened, or attacked in conditions directly related to his/her job and when these situations directly or indirectly endanger his/her safety” [18, p. 113] or involve an explicit or implicit challenge to his/her well-being or health [6].

The literature suggests that the most common sources of violence against healthcare workers are patients and visitors [13]. There are three types of patient and visitor violence: emotional attack, physical violence, and sexual harassment [19]. The term “emotional attack” signifies psychological violence of a verbal, nonphysical nature. While the existence of physical violence in the workplace has always been recognised, the prevalence of psychological violence has long been underestimated and has only recently received due attention [6]. Emotional attacks include verbal abuse, in the form of harsh words, cursing, speaking in an aggressive manner, or a raised voice [18], bullying/mobbing, and written or verbal threats [6, 20] that do not lead to physical injury [1]. Although a single incident can suffice [6], psychological violence is often perpetrated through repeated behaviour of a type that, by in a single incident, may be relatively minor but that, cumulatively, can constitute a serious form of violence [6]. Physical violence can be defined as any form of attack that has a physical component [1, 21] and entails the use of physical force against another person [22]. Sexual harassment is any unwanted, unreciprocated, and unwelcome attention or behaviour of a sexual nature that a person finds offensive and causes that person to feel threatened, unsafe, uncomfortable, humiliated, or embarrassed [22–24].

Violence is significantly underreported in healthcare settings [25], especially in relation to nonphysical forms of violence [26]. The research has provided numerous explanations for the underreporting of violence [20]: feelings of guilt or shame [27], fear of being blamed by the aggressor and/or their family for the violent behaviour or accused of taking revenge [28], a lack of time and unwillingness to fill in forms [29], and concern about consequences [30]. Another reason for this underreporting might be the belief, among some healthcare workers, that they are expected to deal with violence as part of their job [26, 31] and that reporting patient violence conflicts with their duty of care [16]. In some cases, there is a belief that no action will be taken at management level [16, 28, 30].

Recent studies estimate that patient and visitor violence against healthcare workers has been increasing [32, 33], but the lack of systematic investigation [11], the variety of research design used, and the tendency to underreport [34] make it difficult to uncover the true prevalence of violent incidents [35]. Patient and visitor violence towards healthcare workers is a widespread issue [13, 36–39] in both developed and developing countries [32, 40–45]. A recent Italian study [46] confirms the diffusion of patient and visitor violence against healthcare professionals. In another Italian study, the percentage of respondents reporting patient and visitor violence ranged from 48.6 to 65.9 [20]. Ramacciati and colleagues [47] find that in the twelve months prior to the date of data collection 76.0% of emergency nurses experienced verbal violence. A study on Italian hospital staff shows that 60.2% of workers experienced psychological violence; the most frequent type of verbal violence is insulting and shouting. Another result is that 31.5% of hospital staff members experienced physical violence; the most frequent type is pushing [25].

The causes and contributing factors that give rise to these forms of violence have a variety of personal and situational aspects [5, 48]. In the healthcare setting, patients and their visitors are often going through a difficult time [49]. The experience of illness and the processes they have to go through as a result cause fear and anxiety, and these need to be managed [2, 31]. In these conditions, patients and visitors are dependent on healthcare staff [18], and this relationship has a key role in the care process. Problematic interactions between healthcare workers and patients or visitors can increase the likelihood of violence [15]. Person-related factors include poor [10] and rude [15] communication, insufficient communication [31], miscommunication [38], misunderstandings [50], shortcomings in the way information is shared between practitioner and patient [2], lack of trust between doctors and patients [44, 51], unmet or unrealistic expectations on the part of patients and their families [19], dissatisfaction with the course of treatment and/or disagreement with the doctor [1], and poor patient compliance [9]. Important situational factors that contribute to patient and visitor violence include a combination of organisational [15, 17–19, 28, 32, 50] and the environmental conditions [9, 16, 28, 32, 49, 52]. Other factors include loss of respect for the doctor [53] and the perception of a poor standard of care [2, 44].

Many studies on patient and visitor violence recognise that, irrespective of cultural differences and disparate risk factors, the consequences of violence are similar [21]. Both direct and indirect exposure [54] to violence has immediate, extensive [32], and often long-term effects [22] on various levels [55]. Workplace violence is associated with a reduction in job satisfaction. The impact of doctor and nurse satisfaction on patient care has also been investigated: more satisfied healthcare workers have more satisfied patients [56, 57], and low doctor and nurse satisfaction is associated with higher attrition rates [58]. Job satisfaction is defined as the level of contentment a worker feels regarding his or her job [59]. Doctors, as a group, appear to be satisfied with their work, and some of the highest satisfaction scores have been seen in the area of patient relationships [60]. Patient and visitor violence is also associated with increased job stress [30, 35, 61]. When low job satisfaction, stress, and violence intersect at the workplace, as they often do, the negative effects accumulate rapidly and give rise to a vicious cycle, which is very difficult to untangle [22]. In addition to the negative effects mentioned above, violence against healthcare staff can have a negative economic impact [62]. Workplace violence also has an impact on healthcare workers' careers and their ability to make decisions [32] and to carry out their day-to-day duties [12]. It also reduces their commitment to good care practice and undermines their confidence in their own professional capabilities [63] and leads to an increase in errors [32] and, in extreme cases, even malpractice [18]. It is possible that violent encounters contribute to a drop in the quality and efficiency of the entire healthcare system [12]. Another consequence of violent incidents relates to the individuals' attachment system, which is activated by signs of threat [64]. Attachment style has been thought to play a significant role in the healthcare worker-patient relationship [65]. Our attachment style affects both the way we perceive others in terms of threat and the way we perceive any type of interpersonal issue or problem [66]. We believe there is a gap in the literature regarding the way the attachment styles of healthcare workers affect their perceptions of patient and visitor violence.

The aim of our study was to determine the prevalence of violent behaviour in a number of emergency departments in hospitals in northeast Italy and investigate the relationship between violence and certain psychosocial factors, thereby providing a basis for appropriate intervention. Research in many countries, including Italy [20], indicates that workers in all sectors of healthcare are exposed to workplace violence [25] but that psychiatric [67] and emergency departments are the areas at greatest risk of patient and visitor violence [10], due to several factors that involve both patient pathology and the way services are organised and delivered [20]. Emergency departments are the areas that see the highest rates of patient or visitor violence [43, 54, 68]. The emergency ward is characteristically a high-pressure environment across the 24 hours of the day and experiences a high turnover of patients [37]. Patients and their visitors are often in a state of severe anxiety, stress, and fear and have to manage various forms of frustration and long waiting times, all factors that can increase the likelihood of violent behaviour [20]. This study focuses on the experience of healthcare staff (doctors and nurses) with regard to patient and visitor violence. Studies suggest that patients and their relatives or friends are the main perpetrators of violence in the healthcare workplace [21]. Most authors indicate that patients tend to be more violent than visitors [69–71]. However, other studies have suggested that patients' relatives and friends are more often responsible [30]. Therefore, we decided to investigate which types of violence tended to be perpetrated by patients and by visitors.

This study examines the type and frequency of violence experienced by healthcare staff (doctors and nurses). While most research on violence in healthcare workplaces has focused primarily on the experiences of nurses [5], the majority of participants in our study were doctors. For the purposes of the study, patient and visitor violence includes any form of emotional, physical, or sexual violence. Our analysis also investigates certain psychosocial factors, such as attachment style, job satisfaction, and age, as antecedents of emotional violence. It is hoped that this information will be useful to healthcare staff and contribute to improving working practices and ongoing-training programmes. 

*H1*. We assume that doctors perceive lower levels of patient and visitor violence than nurses. 

*H2*. We assume that insecure (especially avoidant) healthcare workers perceive a greater level of violence in the actions and reactions of patients and visitors than secure ones. 

*H3*. We assume that more satisfied healthcare workers perceive lower levels of patient and visitor violence. 

*H4*. We explored the impact of attachment style, age, and job satisfaction on patient and visitor violence. We assume that secure and senior healthcare workers perceive lower levels of patient-and-visitor-perpetrated emotional violence; we also tested the mediating effect of job satisfaction.

## 2. Method

### 2.1. Ethics Statement

The data for this study were collected using an online questionnaire. Ethical approval for the study was guaranteed by the Ethics Committee at the researchers' institution. The questionnaires included a section that explained the nature and purpose of the study and a consent form. All respondents participated voluntarily and provided informed consent. Participants were informed that they could withdraw from the study or refuse to give information at any time without negative consequences of any sort. We preserved the privacy and anonymity of the doctors and nurses involved in the study. The email addresses used to distribute the questionnaires were provided by the managers of the emergency departments involved.

### 2.2. Subjects and Data Collection

Participants completed a questionnaire including measures of patient and visitor violence, attachment style, and job satisfaction. The questionnaires were completed in February 2019. Using email, we contacted 395 nurses and doctors who work in eight emergency departments in northeastern Italy. The research participants were selected on voluntary basis. A total of 149 questionnaires were completed (response rate of 37.73%). The gender distribution was 79 males (53.00%) and 69 females (46.30%); 1 participant did not indicate their gender (0.70%). The ages of the respondents ranged from 24 to 72 (*M* = 46.84;* SD* = 11.36; 2 missing data, 1.34%). The mean length of service was 18.47 years (*SD* = 11.93;* range* = 1-45; 1 missing data, 0.67%). The majority of the participants in this study were doctors (58.40%). A summary of the gender, age, and length-of-service data is reported in Table 1.

### 2.3. Measurement and Data Analysis

The questionnaire included questions on demographic and occupational characteristics, types of violence in the workplace perpetrated by patients and visitors [72, 73], attachment style [74, 75], and job satisfaction [51, 76]. 

*Types of Violence at Work*. We evaluated three different types of violence (emotional, physical, and sexual) [72, 73]. We asked participants about their experiences of violence perpetrated by patients and visitors (patients' relatives or friends). Emotional violence includes verbal abuse, intimidation, obscene behaviour, threatening behaviour, threats, threats made over the telephone, threats to family, slander, and vexatious complaint. Physical violence encompasses property damage or theft, physical abuse, injury, and stalking. Sexual violence comprises inappropriate touching, sexual harassment, and sexual abuse. Responses were given on a 4-point Likert scale, ranging from 1 (*never*) to 4 (*always*). Cronbach's alphas were .883 (9 items,* emotional violence perpetrated by patients*), .699 (4 items,* physical violence perpetrated by patients*), .886 (9 items,* emotional violence perpetrated by visitors*), .754 (4 items,* physical violence perpetrated by visitors*), .928 (18 items,* emotional violence perpetrated by patients and visitors*), and .804 (8 items,* physical violence perpetrated by patients and visitors*). The alpha coefficients for the three items of* patient-perpetrated sexual violence *and the three items of* visitor-perpetrated sexual violence *were below acceptable levels, possibly due to poor interrelatedness between items or due to significant differences in content and type of sexual violence. Therefore, in order to measure* patient-perpetrated sexual violence *and* visitor-perpetrated sexual violence *we used two individual items: “inappropriate touching by patients” and “inappropriate touching by visitors.”

*Attachment style* was evaluated using Adult Attachment Types [74, 75]. For each attachment type (avoidant, anxious, and secure) the participants indicated a level of agreement (or disagreement) with the description of how they typically felt in relationships: avoidant type: “I am some-what uncomfortable being close to others; I find it difficult to trust them completely, difficult to allow myself to depend on them. I am nervous when anyone gets too close, and often, love partners want me to be more intimate than I feel comfortable being”; anxious type: “I find that others are reluctant to get as close as I would like. I often worry that my partner does not really love me or won't stay with me. I want to merge completely with another person, and this desire some-times scares people away”; and secure type: “I find it relatively easy to get close to others and I am comfortable depending on them and having them depend on me. I do not often worry about being abandoned or about someone getting too close to me.” Responses were given on a 4-point Likert scale, ranging from 1 (*completely disagree*) to 4 (*completely agree*). 

*Job Satisfaction. *We used the 5-item* Global Job Satisfaction* scale from the Physician Worklife Survey [51, 76] (e.g., “Overall, I am pleased with my work”). Participants' responses were recorded on a 4-point Likert scale that ranged from 1 (*completely disagree*) to 4 (*completely agree*). Cronbach's alphas were .873.

Data analysis was performed using the SPSS statistical software package. First, for each variable, a composite score was computed by averaging the respective items. Pearson correlation was used to examine the association between variables. Using a paired sample* t*-test we analysed the differences between emotional and physical violence, emotional violence perpetrated by patients and emotional violence perpetrated by visitors, physical violence perpetrated by patients and physical violence perpetrated by visitors, and inappropriate touching by patients and inappropriate touching by visitors. To test whether male and female nurses and doctors reported different levels of perceived patient and visitor violence and job satisfaction independent,* t*-tests were applied. Finally, multiple linear regression analyses were conducted. The regression models included attachment style, job satisfaction, and age as predictors and emotional violence as a dependent variable. We explored the impact of attachment style and age on patient-and-visitor-perpetrated emotional violence. We also tested the effect of job satisfaction as a mediating variable in the relationship between attachment style, age, and patient-and-visitor-perpetrated emotional violence, using the bootstrapping procedure [77]. In accordance with this procedure, three regression analyses were conducted. All regressions were carried out on 5,000 resamples. First, the mediator (job satisfaction) was regressed on the independent variables (anxious attachment, avoidant attachment, secure attachment, and age). Second, the dependent variable (patient-and-visitor-perpetrated emotional violence) was regressed on the independent variables; this second regression equation estimates the total effect of the independent variables on the dependent variables. Finally, the dependent variable was regressed simultaneously on both the mediator and the independent variables; this third regression equation estimates the direct effect. The indirect effect is calculated as the product of the regression coefficients obtained in the first and third regressions. It is significant if zero is not included in the confidence interval [78].

## 3. Results

### 3.1. Descriptive Statistics, Paired Sample, and Independent* t*-Test

The means and standard deviations of the research variables are presented in Table 2. There were differences in the type and frequency of violence experienced by healthcare staff and in the violence perpetrated by patients or by visitors (patients' relatives or friends). Nurses and doctors experienced emotional violence more frequently (*M* = 1.86,* SD* = .55) than physical violence (*M* = 1.20,* SD* = .30;* p* < .001). The results indicate that patients tend to be more violent than visitors: emotional violence is perpetrated more frequently by patients (*M* = 1.97,* SD* = .60) than by visitors (*M* = 1.75,* SD* = .58;* p* < .001); likewise, physical violence is perpetrated more frequently by patients (*M* = 1.30,* SD* = .41) than by visitors (*M* = 1.10,* SD* = .24;* p* <.001), and inappropriate touching is perpetrated more frequently by patients (*M* = 1.26,* SD* = .58) than by visitors (*M* = 1.09,* SD* = .36;* p* < .005). There were also differences between the professions in terms of exposure to patient and visitor violence (Table 3). Nurses reported higher risk of patient and visitor violence than doctors, with specific reference to patient-perpetrated emotional violence (*p* < .001), visitor-perpetrated emotional violence (*p* < .001), patient-and-visitor-perpetrated emotional violence (*p* < .001), patient-perpetrated physical violence (*p* < .020), patient-and-visitor-perpetrated physical violence (*p* < .025), inappropriate touching by patients (*p* < .020), and inappropriate touching by visitors (*p* < .015). We did not identify differences in relation to visitor-perpetrated physical violence. These results support our first hypothesis: doctors perceive lower levels of patient and visitor violence than nurses do. There were no significant gender differences in terms of exposure to patient and visitor violence.

### 3.2. Correlations

In line with our assumptions, the results (Table 2) reveal that avoidant attachment correlates positively with visitor-perpetrated emotional violence and patient-and-visitor-perpetrated emotional violence. Secure attachment, however, correlated negatively with visitor-perpetrated emotional violence, patient-and-visitor-perpetrated emotional violence, and patient-perpetrated physical violence. With regard to sexual violence, the correlations reveal that inappropriate touching by visitors correlated positively with both avoidant and anxious attachment. These results support our second hypothesis. Regarding the other relations between variables, the results indicate that patient-and-visitor-perpetrated emotional violence is negatively correlated with job satisfaction (these results support our third hypothesis) and with age. According to the literature, the perception of patient and visitor violence decreases as the healthcare worker's age increases.

### 3.3. Multiple Regression Analysis of Variables on Patient and Visitor Emotional Violence

Table 4 details the estimates and the 95% bias corrected confidence intervals. The results show that job satisfaction is negatively related to avoidant attachment and positively related to secure attachment. Regarding patient and visitor emotional violence, the results show that its relation with secure attachment and healthcare workers' age is negative. The overall effects of anxious and avoidant attachment were not significant. Regarding job satisfaction, the results indicate that it is negatively related with patient and visitor emotional violence. As for the indirect effects, the results show that job satisfaction mediates the effects of secure attachment; indeed, zero is not included in the confidence interval. Secure healthcare professionals experience lower levels of patient-and-visitor-perpetrated emotional violence, and this can be explained by their level of perceived job satisfaction. These results support our fourth hypothesis.

## 4. Discussion and Conclusions

The aim of our study was to explore the experience of violent acts in a number of emergency departments in Italian hospitals, offering an indication of the prevalence of patient-and-visitor-perpetrated violence and assessing the relationship between violence and psychosocial factors, with a view to providing a basis for appropriate intervention. The results of our study corroborate previous research and confirm existing evidence, also in Italy [20], that patient and visitor violence is a significant risk for healthcare workers. Our study reports different types of violence that healthcare workers experience on a regular basis. In accordance with previous studies, emotional violence is the most prevalent form experienced [5, 11, 43, 63], and patients are the main source of violence against healthcare staff [20, 70, 71].

In line with a number of studies [17, 32, 69], we confirm that there are no statistically significant differences between male and female workers in terms of exposure to patient and visitor violence. According to the literature [30, 47, 55], the age of the healthcare staff has an effect on their experience of patient and visitor violence. Older respondents report experiencing less emotional violence than younger respondents do. It seems that older, and thus more experienced, healthcare workers acquire skills in patient and visitor management and communication and in deescalating confrontational situations. These enable them to defuse cases of verbal violence but may be less helpful in avoiding physical incidents [37, 44, 51, 63]. As expected, exposure to patient and visitor violence is also different among healthcare professions; the professionals at greatest risk of violence are nurses [10, 15, 26, 79] who are more likely to experience (emotional and physical) violence than doctors. This could be explained by many factors: the job of a nurse involves close personal contact with patients [1, 69] and a lot of time spent providing patient care [21, 55]. Furthermore, they often work alone, have access to drugs, and provide care to people in distress. Moreover, patients' perception that doctors have greater seniority than nurses may mean that the former are less likely to be threatened with emotional or physical violence than the latter [20].

We tested the effects of job satisfaction, attachment style, and age on patient and visitor emotional violence. Results of multiple regression analysis suggest an association between emotional violence and age and secure attachment: higher scores in secure attachment and greater age are associated with reduced experience of patient-and-visitor-perpetrated emotional violence. We also tested the function of job satisfaction as a mediating variable: results show that there is no mediating effect in the relationship between age and emotional violence; conversely, job satisfaction mediates the relationship between secure attachment and patient-and-visitor-perpetrated emotional violence. Our results confirm that the attachment style of a healthcare professional influences their perception of patient and visitor violence. According to the literature [66, 80], attachment style is an important variable that profoundly influences the way an individual perceives others and themselves in any type of threatening or violent interpersonal situation. Insecure attached individuals tend to be skeptical of apparent goodwill on the part of others and perceive any type of negative relational event or situation as more threatening than a secure attached person would. This perception is rooted in their thoughts and beliefs about themselves and other people and in the relational strategies they use in managing interpersonal closeness and/or distance. Moreover, secure attached healthcare professionals are more satisfied with their job [81, 82] and find more fulfilment than their insecure attached colleagues in the caring relationship with patients. In addition, insecure medical professionals, when asked to report their sources of well-being at work, cite fewer items in the relatedness area [83]. Insecure individuals, who perceive negative behaviour on the part of others as more threatening than secure people, may be inclined to attribute other people with hostile intentions [84, 85].

Our results support the conclusion that job satisfaction is the best predictor of patient-and-visitor-perpetrated emotional violence. Different researchers associate dissatisfaction among healthcare staff with an increased likelihood of violence. This is probably because of differences in the approach that dissatisfied doctors and nurses take to conflict, the deleterious effect of their dissatisfaction on the practitioner-patient relationship [51], and the lower standards of care that they are able to provide [86]. When violence and low job satisfaction interact, their negative effects cumulate rapidly and give rise to a vicious cycle [22]. There is a close association between patient and visitor violence and low job satisfaction, but the direction of this relationship is not entirely clear [35], that is, whether violence causes a reduction in job satisfaction or if dissatisfaction among workers makes them more likely to be victims of violence. In accordance with findings reported in the literature [87, 88], our results suggest that doctors and nurses are at the greatest risk for violence when they are dissatisfied with their job and that the promotion of well-being at work and job satisfaction could effectively contribute to coping with, and preventing, patient and visitor violence [6].

For a variety of reasons, it is unlikely that violence in healthcare workplaces can be completely eliminated [28]. Prevention strategies and interventions are key to reducing future violence [50, 54]. Healthcare staff should therefore be trained to recognise and prevent violence and/or to manage violent situations adequately [68], because they are often not able to foresee a violent act and do not have any premonition of danger before being assaulted [20]. Moreover, training will help healthcare staff developing coping strategies [7, 32, 89] to identify, understand, and manage risk of patient and visitor violence. Our study confirms that emotional violence is the most frequent form of violence. As such, it is important to provide staff with training [9, 21, 90] to improve communication skills for calming patients and visitors [5, 19], adopt deescalation strategies [17], and develop capabilities in conflict resolution [10]. Our results also suggest that training interventions should be accompanied by efforts to improve job satisfaction. A second aspect to consider in preventing patient and visitor violence is the implementation of a safe practice environment [32, 54, 72]. Changes to the work environment and at an organisation level, for example, could include fixed emergency alarms and security guards [50], but there is also scope for improvements to occupational health and safety legislation and policy [17, 69]. Teamwork and a supportive workplace can further be helpful and effective in this regard [10, 20, 21].

We can list a number of limitations to our study. First, since our research was limited to a small number of emergency departments, we cannot generalise our findings to all Italian emergency departments. However, our results agree with the literature, and we have no evidence to suggest that the situation is different in other emergency departments. Second, our study used a cross-sectional research design. The selection of subjects therefore introduced an expected bias and no conclusions can be drawn as to the causality of the relationships between variables. A longitudinal study would be desirable at this point. Third, there is a relatively small number of healthcare workers that completed our questionnaire. The response rate (37.73%) represents a satisfactory outcome. We did not expect that every healthcare professional who received the questionnaire would complete it: workplace violence can be an embarrassing subject and difficult to report, and nurses and doctors are very busy. Fourth, the study used a retrospective self-reporting approach for data collection. This method depends on the ability of the participants to recall events from the 12 months prior to collecting the data, which might be affected by recall bias. However, as most studies in the field of patient and visitor violence use a 12-month self-assessment time frame [15, 69], this procedure is important in enabling comparisons. Finally, the present study relied on self-reported measurements. However, to minimise problems, we used validated questionnaires that have been shown to have good reliability.

Future research should aim to investigate the relationship between patients' and visitors' perception of violence and healthcare staff's levels of job stress. According to Ferri and colleagues [20], in order to be able to suggest effective actions to prevent violence, future studies should explore collaboration practices and perceived social support among healthcare staff members. Additional research should focus on comparing different departments selected according to their differences in violent behaviour incidence.

## Figures and Tables

**Table 1:** Participants' characteristics (*N* = 149).

Variables	Whole Sample		Physicians		Nurses	
Gender						
Males	79	(53.0%)	50	(57.5%)	29	(46.8%)
Females	69	(46.3%)	37	(42.5%)	32	(51.6%)
Missing value	1	(0.7%)	0	(0%)	1	(1,6%)
Age						
24 to 30 years	11	(7.4%)	3	(3.4%)	8	(12.9%)
31 to 40 years	38	(25.5%)	26	(29.9%)	12	(19.4%)
41 to 50 years	43	(28.9%)	12	(13.8%)	31	(50%)
51 to 60 years	36	(24.2%)	26	(29.9%)	10	(16.1%)
Over 60 years	19	(12.8%)	19	(21.8%)	0	(0%)
Missing value	2	(1.3%)	1	(1.1%)	1	(1.6%)
Length of service						
1 to 10 years	51	(34.2%)	34	39.1%)	17	(27.4%)
11 to 20 years	24	(16.1%)	15	(17.2%)	9	14.5%)
21 to 30 years	54	(36.2%)	23	(26.4%)	31	(50%)
31 to 40 years	19	(12.8%)	15	(17.2%)	4	(6.5%)
Missing value	1	(0.7%)	0	(0%)	1	(1.6%)

**Table 2:** Descriptive Statistics and Intercorrelations (*N* = 149).

		Mean	SD	1	2	3	4	5	6	7	8	9	10	11	12
1	Emotional Violence by Patients	1.97	.60	-											

2	Emotional Violence by Visitors	1.75	.58	.733*∗∗∗*	-										

3	Emotional Violence by Patients and Visitors	1.86	.55	.934*∗∗∗*	.928*∗∗∗*	-									

4	Physical Violence by Patients	1.30	.41	.631*∗∗∗*	.517*∗∗∗*	.618*∗∗∗*	-								

5	Physical Violence by Visitors	1.10	.24	.520*∗∗∗*	.515*∗∗∗*	.556*∗∗∗*	.650*∗∗∗*	-							

6	Physical Violence by Patients and Visitors	1.20	.30	.645*∗∗∗*	.565*∗∗∗*	.651*∗∗∗*	.951*∗∗∗*	.852*∗∗∗*	-						

7	Inappropriate Touching by Patients	1.26	.58	.299*∗∗∗*	.285*∗∗∗*	.314*∗∗∗*	.344*∗∗∗*	.287*∗∗∗*	.353*∗∗∗*	-					

8	Inappropriate Touching by Visitors	1.09	.36	.358*∗∗∗*	.330*∗∗∗*	.370*∗∗∗*	.353*∗∗∗*	.166*∗*	.310*∗∗∗*	.373*∗∗∗*	-				

9	Secure Attachment	2.45	.95	-.211*∗∗*	-.110	-.173*∗*	-.173*∗*	-.059	-.143	.036	-.086	-			

10	Avoidant Attachment	1.68	.74	.149	.213*∗∗*	.194*∗*	.132	.018	.099	.126	.217*∗∗*	-.173*∗*	-		

11	Anxious Attachment	1.41	.62	.094	.021	.062	.005	-.006	.001	-.067	.194*∗*	-.132	.272*∗∗*	-	

12	Global Job Satisfaction	2.97	.75	-.188*∗*	-.162*∗*	-.188*∗*	.015	.022	.019	.022	-.228*∗∗*	.229*∗∗*	-.218*∗∗*	-.156	-

13	Age	46.84	11.36	-.160	-.262*∗∗*	-.225*∗∗*	-.085	-.148	-.118	-.172*∗*	-.089	-.078	-.097	.138	-.151

*∗p*<0.05, *∗∗p*<0.01, and *∗∗∗p*<0.001.

**Table 3:** Differences in the sample means (*N* = 149).

		Men	Women	t	Physicians	Nurses	t
1	Emotional Violence by Patients	1.90	2.04	1.41	1.76	2.26	5.47*∗∗∗*
		(.58)	(.61)		(.51)	(.60)	

2	Emotional Violence by Visitors	1.66	1.85	2.01*∗*	1.54	2.03	5.65*∗∗∗*
		(.56)	(.59)		(.49)	(.57)	

3	Emotional Violence by Patients and Visitors	1.78	1.94	1.83	1.65	2.15	5.87*∗∗∗*
		(.52)	(.57)		(.45)	(.55)	

4	Physical Violence by Patients	1.29	1.31	.30	1.23	1.40	2.44*∗*
		(.39)	(.43)		(.41)	(.39)	

5	Physical Violence by Visitors	1.08	1.12	.94	1.07	1.14	1.58
		(.20)	(.28)		(.24)	(.23)	

6	Physical Violence by Patients and Visitors	1.19	1.22	.59	1.15	1.27	2.32*∗*
		(.26)	(.33)		(.30)	(.27)	

7	Inappropriate Touching by Patients	1.22	1.30	.93	1.15	1.40	2.42*∗*
		(.57)	(.60)		(.39)	(.76)	

8	Inappropriate Touching by Visitors	1.13	1.06	-.1.21	1.02	1.19	2.57*∗*
		(.44)	(.24)		(.15)	(.51)	

9	Secure Attachment	2.43	2.48	.31	2.62	2.21	-2.66*∗*
		(.98)	(.92)		(.94)	(.91)	

10	Avoidant Attachment	1.59	1.77	1.44	1.56	1.85	2.42*∗*
		(.73)	(.73)		(.68)	(.79)	

11	Anxious Attachment	1.46	1.35	-.1.06	1.39	1.44	.44
		(.66)	(.56)		(.64)	(.59)	

12	Global Job Satisfaction	2.95	3.00	.41	3.01	2.91	-.80
		(.75)	(.75)		(.74)	(.76)	

13	Age	49.14	44.21	-2.66*∗*	49.51	43.08	-3.69*∗∗∗*
		(11.69)	(10.53)		(12.15)	(8.96)	

*∗p*<0.05, *∗∗p*<0.01, and *∗∗∗p*<0.001.

**Table 4:** Mediation effects of global job satisfaction on patients and visitors' emotional violence (*N* = 149).

		Global Job Satisfaction	Patients and Visitors' Emotional Violence		Indirect effect	Bias correct 95% confidence interval	
		*β*	*β*	*β*	*β*	Lower	Upper
		(*SE*)	(*SE*)	(*SE*)	(*SE*)		
1	Secure Attachment	.141*∗*	-.099*∗*	-.080	-.019	-.192	-.003
		(.064)	(.047)	(.047)			

2	Avoidant Attachment	-.192*∗*	.098	.073	.026	-.034	.238
		(.085)	(.062)	(.062)			

3	Anxious Attachment	-.081	.023	.012	.011	-.141	.186
		(.101)	(.074)	(.073)			

4	Age	-.010	-.011*∗∗*	-.012*∗∗*	.001	-.018	-.004
		(.005)	(.004)	(.004)			

5	Global Job Satisfaction			-.133*∗*			
				(.061)			

	*R* ^*2*^	.116	.109	.138			

	*F*	4.639*∗∗*	4.333*∗∗*	4.519*∗∗*			

	*df*	4,142	4,142	5,141			

*β* = unstandardized coefficient; 5,000 resamples.

*∗p*<0.05, *∗∗p*<0.01, and *∗∗∗p*<0.001.

## Data Availability

The dataset used to support the findings of this study are available from the corresponding author upon request.
